# Inotuzumab ozogamicin in pediatric patients with relapsed/refractory acute lymphoblastic leukemia

**DOI:** 10.1038/s41375-018-0265-z

**Published:** 2018-09-28

**Authors:** Deepa Bhojwani, Richard Sposto, Nirali N. Shah, Vilmarie Rodriguez, Constance Yuan, Maryalice Stetler-Stevenson, Maureen M. O’Brien, Jennifer L. McNeer, Amrana Quereshi, Aurelie Cabannes, Paul Schlegel, Claudia Rossig, Luciano Dalla-Pozza, Keith August, Sarah Alexander, Jean-Pierre Bourquin, Michel Zwaan, Elizabeth A. Raetz, Mignon L. Loh, Susan R. Rheingold

**Affiliations:** 1 Children’s Hospital Los Angeles and Keck School of Medicine, University of Southern California, Los Angeles, CA USA; 2 National Cancer Institute, Bethesda, MD USA; 3 Mayo Clinic, Rochester, MN USA; 4 Cincinnati Children’s Hospital Medical Center, University of Cincinnati School of Medicine, Cincinnati, OH USA; 5 University of Chicago Medicine Comer Children’s Hospital, Chicago, IL USA; 6 Oxford University Hospitals, Oxford, UK; 7 University Hospital Saint-Louis, Paris, France; 8 University Children’s Hospital of Würzburg, Würzburg, Germany; 9 University Children’s Hospital of Münster, Münster, Germany; 10 Children’s Hospital at West-mead, Sydney, Australia; 11 Children’s Mercy Hospital, Kansas City, MO, USA; 12 The Hospital for Sick Children, Toronto, Canada; 13 University Children’s Hospital, Zurich, Switzerland; 14 Erasmus MC, University Medical Center, Rotterdam, the Netherlands; 15 New York University Langone Medical Center, New York, NY USA; 16 Benioff Children’s Hospital and the Helen Diller Family Comprehensive Cancer Center, University of California San Francisco, San Francisco, CA USA; 17 Childrens Hospital of Philadelphia, University of Pennsylvania, Philadelphia, PA USA

**Keywords:** Cancer immunotherapy, Acute lymphocytic leukaemia

## Abstract

Although inotuzumab ozogamicin (InO) is recognized as an effective agent in relapsed acute lymphoblastic leukemia (ALL) in adults, data on safety and efficacy in pediatric patients are scarce. We report the use of InO in 51 children with relapsed/refractory ALL treated in the compassionate use program. In this heavily pretreated cohort, complete remission was achieved in 67% of patients with overt marrow disease. The majority (71%) of responders were negative for minimal residual disease. Responses were observed irrespective of cytogenetic subtype or number or type of prior treatment regimens. InO was well-tolerated; grade 3 hepatic transaminitis or hyperbilirubinemia were noted in 6 (12%) and grade 3/4 infections in 11 (22%) patients. No patient developed sinusoidal obstruction syndrome (SOS) during InO therapy; however, 11 of 21 (52%) patients who underwent hematopoietic stem cell transplantation (HSCT) following InO developed SOS. Downregulation of surface CD22 was detected as a possible escape mechanism in three patients who developed a subsequent relapse after InO. We conclude that InO is a well-tolerated, effective therapy for children with relapsed ALL and prospective studies are warranted. Identification of risk factors for developing post-HSCT SOS and strategies to mitigate this risk are ongoing.

## Introduction

Despite tremendous progress made in the therapy for childhood acute lymphoblastic leukemia (ALL), approximately 10–15% of children relapse [1]. Intensive salvage regimens result in suboptimal 2-year disease-free survival rates of only 41 and 13% for children in second and third remission respectively [2]. Furthermore, these outcomes have not improved significantly in the past two decades [3]. Progressive leukemia remains one of the leading causes of disease-related death in children and novel therapeutic options are urgently needed.

Inotuzumab ozogamicin (InO) is a CD22-directed humanized monoclonal antibody conjugated to the potent cytotoxin calicheamicin [4]. CD22 is widely expressed on B-ALL blasts and is rapidly internalized upon antibody binding, making it an excellent target for immune-targeted chemotherapy in B-ALL [5, 6]. In studies of adult patients with relapsed/refractory B-ALL, InO has demonstrated impressive single agent activity [4, 7]. The overall response rate in a phase II study of 49 patients was 58% [8]. Of patients who responded, 72% achieved minimal residual disease (MRD) negativity (<0.01%). A fractionated dosing regimen was associated with less hepatotoxicity than a single dose regimen and was studied in a large randomized phase 3 study (INO-VATE) wherein patients were randomized to receive InO vs. standard chemotherapy for first or second salvage [9]. The complete response (CR) rate for the InO group was 80.7% compared to 33.3% for the standard of care group (*p* < 0.0001). MRD-negativity was achieved at a higher rate in the InO arm (78.4 vs. 28.1%, *p* < 0.0001) and duration of remission was greater. Based on the results of this study, InO was granted Breakthrough Therapy designation by the U.S. Food and Drug Administration (FDA) in October 2015, and was approved by the FDA in August 2017 for the treatment of relapsed/refractory B-ALL in adults [10].

InO is relatively well tolerated in adult patients. However, similar to the experience with gemtuzumab ozogamicin, an anti-CD33 antibody drug conjugate also linked to calicheamicin, an increased incidence of sinusoidal obstruction syndrome (SOS), previously known as veno-occlusive disease, has been observed with InO [11, 12]. InO-associated SOS is particularly notable in the post-hematopoietic stem cell transplant (HSCT) setting. Other common toxicities observed are thrombocytopenia, neutropenia, fever, liver function abnormalities, and diarrhea [9]. Several studies in adult patients that combine InO with conventional chemotherapy in frontline and relapsed ALL have been recently reported [13, 14].

Leukemic blasts from children with B-ALL strongly express CD22 [6], but pediatric experience with InO is extremely limited, with one phase II adult trial having also enrolled five children [8, 15]. Three patients received the single dose regimen, and two received the fractionated dose regimen. Three of these five patients achieved a CR and had a comparable toxicity profile to the adult cohort, including fever, sepsis and hepatic transaminase elevation. In spite of remarkable efficacy in relapsed/refractory disease and the FDA approval of InO in adults, development of this agent in children has lagged, with the first pediatric studies initiated in 2016–2017. In the interim, Pfizer provided InO to approximately 100 children from 2013 to 2016 via a compassionate use program. In this study, we collected retrospective data from multiple international pediatric oncology centers that treated children with InO by compassionate use and performed a detailed review of the activity and toxicity profile of InO in children with relapsed/refractory ALL. Data from this study will inform larger prospective pediatric studies and provide guidance regarding InO therapy and anticipated toxicity to clinicians awaiting results of pediatric clinical trials.

## Subjects and methods

This was a retrospective cohort study of pediatric patients with relapsed/refractory B-ALL who received InO in the compassionate use program. Eligibility criteria for this program included unavailability of satisfactory alternate therapy, CD22 positivity on leukemic blasts, serum creatinine ≤ 1.5×, bilirubin ≤ 2× and AST/ALT ≤ 2.5× upper limit of normal, absence of severe uncontrolled infection, no history of prior SOS, and a washout of 14 days from another investigational agent. Patients/families consented to monitoring of toxicities.

This current study was not linked to Pfizer or regulatory approvals for drug procurement or administration. Participating pediatric oncology centers obtained separate local institution review board (IRB) or ethics committee approvals as applicable, to contribute demographic, treatment, outcome and toxicity data to this study. Participating physicians and research coordinators reviewed patient medical records to collect data and sent de-identified data to the study team for review and analyses.

Patients were eligible if they were ≤21 years of age at the time of InO administration and had received a minimum of one dose of InO. All patients received the now FDA-approved fractionated dosing schedule of InO. One cycle consisted of three doses: 0.8 mg/m^2^ on week 1 followed by 0.5 mg/m^2^ on weeks 2 and 3. One patient with MRD-only disease received 0.5 mg/m^2^/dose for all three doses. In second and subsequent cycles, assuming CR/CRi during cycle 1, patients received 0.5 mg/m^2^/dose on days 1, 8, and 15. Disease response was determined by local institutions at the completion of each cycle at approximately day 28. Complete remission (CR) was defined as <5% bone marrow blasts by morphology, CR with incomplete count recovery (CRi) as CR with absolute neutrophil count <500/cu.mm or platelet count <50,000/cu.mm. Partial response was defined as the reduction of marrow disease burden from >25% blasts (M3) to 5–25% blasts (M2). MRD was measured either by flow cytometry or polymerase chain reaction (PCR) of immunoglobulin gene rearrangements. Toxicities during and for 30 days post InO therapy were graded per Common Terminology Criteria for Adverse Events (CTCAE) version 4.03. Information from safety reporting forms originally submitted to the sponsor was also collected and included in the analyses. Additional clinical information was gathered for patients who underwent HSCT after treatment with InO to capture occurrence and severity of post-HSCT SOS. Severity of SOS was graded per institutional practice.

### Statistical analyses

Analytic endpoints included CR, event-free survival (EFS) defined as the time from start of treatment to earliest occurrence of treatment failure, disease relapse, or death from any cause, and overall survival (OS) defined as the time from start of treatment to death from any cause. The latter two endpoints were censored at the time of last reported follow-up. The data cutoff date was December 31, 2016. Univariable and multivariable logistic regression was used to examine associations between patient and disease characteristics and the probability of achieving CR. Univariable and multivariable Cox regression analysis was used to assess the associations between EFS/OS and patient/disease characteristics. Estimates of EFS or OS probability were based on the product limit estimator with Greenwood standard errors. Reported *p* values are all two-sided. All analyses were performed using Stata (StataCorp. 2015. Stata Statistical Software: Release 14. College Station, TX: StataCorp LP).

### CD22 antigen expression

In a limited number of patients, there was an opportunity to serially evaluate CD22 expression using methods that have been previously described [6]. Patients were separately consented to another IRB-approved prospective study also targeting CD22 which allowed for screening of their leukemia samples. In this screening process, samples were available for three patients before and after InO without any intervening therapy, allowing for assessment of the impact of InO on CD22 expression.

## Results

### Patient characteristics and InO therapy

From the approximately 100 children treated via the compassionate access program, we obtained data on 51 patients, ages 2.2 years to 21.3 years (median 11.5 years) who received InO from January 2013 to December 2016 at 30 pediatric oncology centers in North America, Europe, and Australia. All but three patients received InO in 2015 or 2016. Detailed patient characteristics are listed in Table 1. Four patients had Down syndrome. Patients were either refractory to primary therapy (*N* = 1) or in first to fifth relapse, and heavily pretreated (2–9 prior regimens; median 5). All ten patients in first relapse had received multiple salvage regimens, including HSCT in four. The majority of patients (*N* = 41, 80%) were refractory to their preceding regimen. Twenty-two patients (43%) had undergone one or more prior HSCT, 40 (78%) had received prior CD19-directed therapy with either CD19 CAR T cells and/or blinatumomab while 10 (20%) had received prior CD22-directed therapy with either CD22 CAR T cells and/or moxetumomab. One patient had received InO on the adult phase II study a year earlier, achieved CR and subsequently relapsed after HSCT. At the time of InO administration, leukemia burden was M3 marrow (>25% blasts) in 38 patients, M2 (5–25% blasts) in 4, M1 ( < 5% blasts) in 8, and unknown in 1 patient. Of eight patients with M1 marrow, all had detectable MRD ranging from 0.05 to 4%. Two patients had concomitant bulky extramedullary disease (salivary gland and a skull-based lesion). The median number of doses of InO received was 5 (range 1–15). Twenty-three (45%) patients received ≤1 cycle, 19 (37%) patients received all or part of two cycles, and 9 (18%) patients received more than two cycles of InO.

**Table 1:** Patient characteristics

Patient characteristics		*N*	%
Location	North America	30	58.8
	Europe	18	35.3
	Australia	3	5.9
Age	2–4 years	3	6
	5–9 years	13	25
	10–17 years	31	61
	18–21 years	4	8
Sex	Male	30	59
	Female	21	41
Down syndrome	Yes	4	8
	No	47	92
Cytogenetic subtype	*ETV6-RUNX1*	5	10
	Hyperdiploid	4	8
	Ph-like	4	8
	Ph-positive	3	6
	Hypodiploid	3	6
	*TCF3-PBX1*	2	4
	*KMT2A*-rearranged	1	2
	t(17;19)	1	2
	iAMP21	1	2
	NOS	19	37
	Unknown	8	16
Indication for InO	First relapse (refractory)	10	20
	Second relapse	22	43
	Third relapse	10	20
	Forth relapse	6	12
	Fifth relapse	2	4
	Primary refractory	1	2
Refractory to preceding regimen	Yes	41	80
	No	9	18
	Unknown	1	2
Number of prior treatment regimens (excluding HSCT)	2–3	8	16
	4–6	28	55
	≥7	15	29
Prior HSCT	None	29	57
	1	18	35
	2	3	6
	3	1	2
Prior CD19-directed therapy	Blinatumomab	22	43
	CD19 CAR T-cells	15	29
	Both of the above	3	6
	None	11	22
Prior CD22-directed therapy	Moxetumomab	6	12
	CD 22 CAR T-cells	3	6
	Both of the above	1	2
	InO	1	2
	None	40	78
Bone marrow status	M1, MRD positive	8	16
	M2	4	8
	M3	38	75
	Unknown	1	1
Extramedullary disease	Yes	2	4
	No	49	96

### Response to InO

Complete responses were reported in 28 of 42 (67%) patients with overt relapse (M2/M3 marrow): CR in 15 (36%) and CRi in 13 (31%). Three patients had a partial response (7%) and eight had no response (19%). Three patients were not evaluable for response as InO was stopped prior to the completion of cycle 1, i.e. less than three doses. Of 28 patients in CR/CRi, 20 (71%) achieved MRD negativity defined as less than 0.01% by flow cytometry or PCR. The majority of patients who achieved CR/CRi (*N* = 24, 86%) did so after the first cycle of InO. All four patients with M2 disease burden achieved MRD-negative CR/CRi. Responses in patients with MRD-positive disease-only (M1 marrow; *N* = 8) were as follows: four achieved MRD negativity, MRD decreased by 1–2 logs in two patients, MRD remained stable in one patient and MRD increased in one patient. The patient with salivary gland disease attained CR in the extramedullary site, with stable marrow MRD, and the patient with a skull-based lesion developed progressive disease at the extramedullary site (but CRi in the marrow). A renal chloroma and vertebral mass were incidentally discovered in one additional patient while receiving InO (unknown if present prior to InO therapy).

No baseline patient or disease characteristic (age, sex, cytogenetic subtype) was identified as a significant prognostic indicator for response. All three patients with Philadelphia chromosome-positive (Ph-positive) ALL attained CR/CRi, one of whom was MRD-negative. Three of four patients with Philadelphia chromosome-like (Ph-like) ALL achieved CR/CRi; one was MRD-negative. Of three patients with hypodiplod ALL, two were in CR post InO (both MRD-positive). The single patient with *KMT2A* rearrangement in this cohort responded well and achieved MRD-negative CR. Three patients with Down syndrome and overt relapse all achieved MRD-negative CR/CRi, and the fourth patient with Down syndrome had a decline in MRD from 4 to 0.5%.

Response to InO was independent of number of prior relapses, number of prior treatment attempts including HSCT, CD19- and CD22-directed immunotherapy, or history of refractoriness to the immediate preceding treatment attempt. Eighteen patients with overt relapse had undergone one or more prior HSCT; all of these patients achieved CR/CRi with InO. Of 11 patients with prior CD22-directed therapy, seven responded well to InO (CR/CRi).

### Post InO therapy and outcome

Twenty-one patients underwent HSCT after InO therapy. The median time from last dose of InO to stem cell infusion was 26 days (range 13–91 days). Two patients received blinatumomab and one received CD19 CAR T-cell therapy as a bridge to HSCT post InO for MRD positivity.

**Fig. 1:**
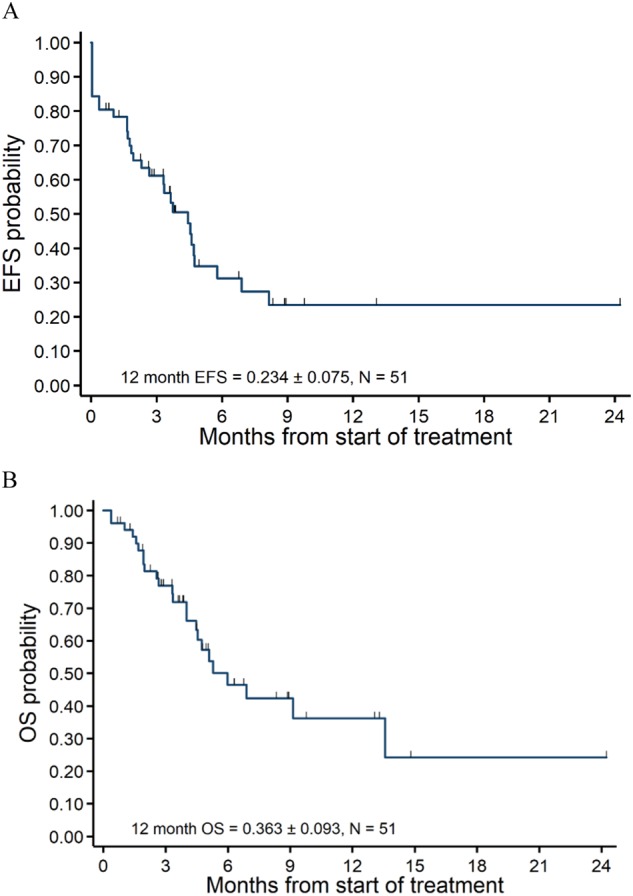
EFS (**a**) and OS (**b**). The 12-month EFS and OS rates for the entire cohort of 51 patients were 23.4 ± 7.5% and 36.3 ± 9.3%, respectively

Thirty-one patients experienced the following first events: eight treatment failures, 12 relapses (includes 4 after HSCT), and 11 deaths (five with disease, six in remission). Median follow-up was 112.5 days in the 20 patients without an event, and 137 days in the 27 patients who were alive at last contact (for both, range 19–736 days). Over 75% of patients were followed for at least 82 days. Sites of relapse in the 12 patients who developed disease recurrence post InO were marrow in ten and isolated extramedullary disease in two patients (central nervous system (CNS) and an extracranial mass in one patient, infiltration of ocular muscle, kidney and pancreas in one patient). Overall, 18 (35%) patients were alive in CR and nine (18%) were alive with evidence of disease. Seven patients (14%) died from transplant-related toxicity while 17 (33%) expired from progressive leukemia. The 12-month EFS and OS rates for the entire cohort were 23.4 ± 7.5% and 36.3 ± 9.3% respectively (Fig. 1).

### CD22 expression post InO

**Fig. 2:**
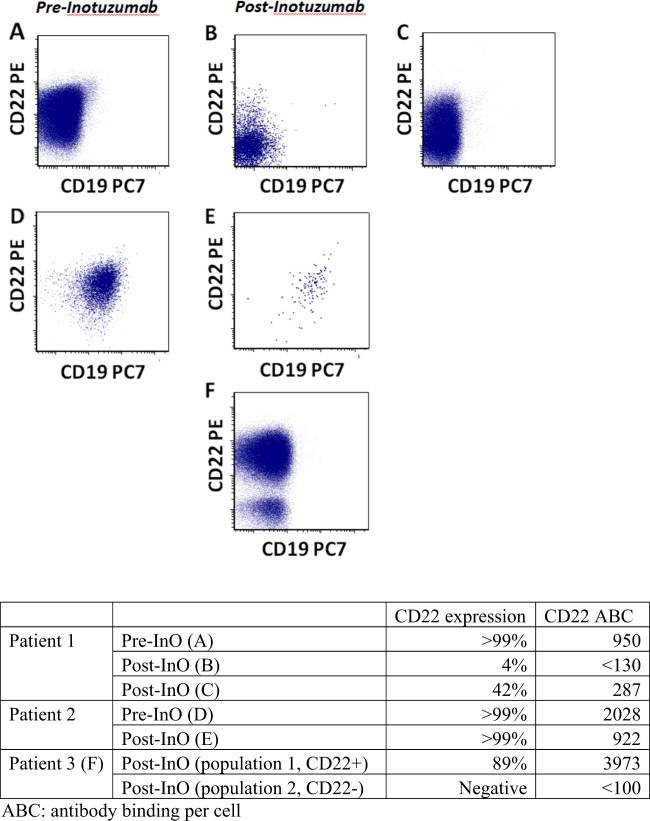
CD22 expression at relapse post-InO. CD22 expression in two patients evaluated pre- and post- InO and in one patient post-InO. CD22 is uniformly expressed on >99% B-lymphoblastic leukemia cells prior to InO (**a**, **d**); however, CD22 expression is diminished or absent (**b**, **c**, **e**) or absent in a subset of lymphoblasts (**f**) after InO

Comprehensive CD22 expression was evaluated in three patients. Pre/post InO leukemia samples were available for two patients, and only a post-InO sample following relapse was available for one patient. In all three cases at the time of relapse, leukemic blasts were noted to be either partial CD22 positive, fully CD22 negative, or had diminution of CD22 expression (Fig. 2). In one case, CD22 expression increased with additional time post InO, but only partial positivity was maintained.

### Toxicities of InO

No patient died from toxicity during InO therapy. One or more nonhematologic toxicities were reported in 34 patients during all cycles. The majority of toxicities (79%) were reported during cycle 1 and are listed in Table 2. Grade 3/4 hepatotoxicity was infrequent, and no patient developed SOS while receiving InO. During the first cycle of InO, one patient developed grade 3 hyperbilirubinemia, while grade 3 ALT and AST elevations were seen in three (6%) and two (4%) patients respectively. In subsequent cycles, only one patient developed recurrent grade 1 AST and ALT elevation and one additional patient developed grade 1 AST elevation. There was no evidence to suggest increased hepatotoxicity with cumulative exposure to InO. Two (4%) patients developed transient grade 3 InO-related infusion reactions with hypotension in cycle 1 and one patient in cycle 2. In this heavily pretreated patient population, febrile neutropenia and infectious toxicities were reported in eight (16%) and 15 (29%) patients respectively (cycle 1 data only). Infectious toxicities included sepsis (*N* = 3), bacteremia (*N* = 2), invasive fungal infections (*N* = 2; both grade 3, one candidemia and one probable lung infection), gastrointestinal (*N* = 3), lung (*N* = 1), sinus (*N* = 1), and unspecified (*N* = 3). Two patients developed bleeding complications (gastrointestinal, epistaxis) while thrombocytopenic. In addition, two patients developed CNS bleeding in the setting of progressive CNS disease during cycle 2. Interestingly bone pain was reported by four (8%) patients in cycle 1 and an additional patient in cycle 2; however, it was not possible to discern if pain was attributable to InO or progressive disease.

**Table 2:** Toxicities during cycle 1

Toxicity	Grade 1–2	Grade 3^a^	Grade 4	Unknown grade	Total
ALT increase	6 (11.8%)	3 (5.9%)			9 (17.6%)
AST increase	8 (15.7%)	2 (3.9%)			10 (19.6%)
GGT increase	2 (3.9%)	1 (2.0%)	1 (2.0%)		4 (7.8%)
Hyperbilirubinemia		1 (2.0%)			1 (2.0%)
Fever	9 (17.6%)				9 (17.6%)
Febrile neutropenia	2 (3.9%)	6 (11.8%)			8 (15.7%)
Infection	4 (7.8%)	8 (15.7%)	2 (3.9%)	1 (2.0%)	15 (29.4%)
Bone pain	3 (5.9%)	1 (2.0%)			4 (7.8%)
Infusion reaction		2 (3.9%)			2 (3.9%)
Vomiting	2 (3.9%)			1 (2.0%)	3 (5.9%)
Diarrhea	1 (2.0%)			1 (2.0%)	2 (3.9%)
Tumor lysis syndrome		2 (3.9%)			2 (3.9%)
Bleeding	1 (2.0%)	1 (2.0%)			2 (3.9%)
Electrolyte disturbances	7 (13.7%)	3 (5.9%)^b^			10 (19.6%)

^a^Additional grade 3 toxicities noted in one patient each: anorexia, hypertension, hypertriglyceridemia, paroxysmal atrial tachycardia

^b^Grade 3 electrolyte disturbances: hypokalemia (2), hypocalcemia (1)

### Post-HSCT sinusoidal obstruction syndrome

Twenty-one patients underwent HSCT after achieving CR. Eleven of 21 patients (52%) developed post-HSCT SOS: four mild, two moderate and five severe, including two events that were fatal. The overall rate of SOS in the entire cohort of 51 patients was 22%. There was a trend to greater risk of SOS in patients who had received one or more HSCT prior to InO: 6/11 (55%) vs. 3/10 (30%) in patients with no prior HSCT, and in patients whose conditioning regimens contained busulfan or clofarabine (Table 3). However, no other potential risk factors, including number of doses of InO, time from InO to HSCT, or conditioning with dual alkylator or total body irradiation were significantly associated with higher incidence of SOS.

**Table 3:** Risk factors for post-transplant SOS

Risk factor	SOS (*N* = 11)	No SOS (*N* = 10)
Median age in years (range)	12 (2–19)	12.5 (7–19)
Median doses of InO (range)	6 (3–12)	4.5 (3–6)
Median days from InO to HSCT (range)	25 (13–91)	30 (13–89)
One or more HSCT prior to InO	6 (55%)	3 (30%)
Dual alkylator conditioning	6 (44%)	7 (54%)^a^
Busulfan containing conditioning	5 (45%)	1 (11%)^a^
Clofarabine containing conditioning	3 (27%)	1 (11%)^a^
TBI conditioning	9 (82%)	6 (60%)^a^

^a^Conditioning regimen was unknown for one patient

## Discussion

As in adults, weekly InO was highly effective in children with relapsed/refractory ALL with a CR/CRi rate of 67%. The majority of these responders (71%) attained MRD negativity. Responses were seen in patients irrespective of disease burden or number and type of prior therapies. We did not identify any patient-specific or disease-related factors that predicted for effectiveness of InO. Encouraging responses were noted in patients with high-risk features such as Ph-positive, Ph-like and hypodiploid ALL, as well as in patients with Down syndrome. Forty percent of patients were able to proceed to HSCT with curative intent.

Within the relatively short follow-up period, 12 patients developed a subsequent relapse post InO. Though we only quantified CD22 expression for three patients in this cohort, one important escape mechanism at relapse may be modulation of the CD22 antigen expression on leukemic blasts, analogous to antigen loss associated with CD19-directed therapies such as blinatumomab and CD19-CAR T-cell therapies [16, 17]. Similar analyses on a larger number of patients will provide further insights into resistance to CD22-directed therapy.

InO was generally tolerated well, even by patients who were heavily pretreated. Infectious toxicity was lower than what is typically reported with chemotherapy regimens for patients with multiply relapsed ALL [18, 19]. Hyperbilirubinemia and transaminitis were low-grade and manageable. Hematologic toxicity could not be assessed adequately in this cohort of patients due to baseline cytopenias. In view of fewer infectious and organ toxicities compared to intensive cytotoxic chemotherapy, InO like other novel immune-targeted therapies, is an attractive means to achieve remission in a patient with relapsed/refractory disease to allow for HSCT.

Whereas some adults developed SOS during InO therapy, pediatric patients receiving InO only developed SOS following HSCT. However, the incidence of SOS was high in this pediatric cohort with an overall rate of 22, and 52% in the HSCT setting, vs. 13 and 22% respectively reported in adults in the phase III INO-VATE global trial [12]. Pediatric patients in this study may have had additional independent risk factors for SOS compared to patients enrolled on the adult phase II study wherein the incidence of post-HSCT SOS was 23% [8]. As this was a compassionate use study, all patients were heavily pretreated with multiple intensive chemotherapy and immunotherapy regimens; 92% of patients received InO for ≥3rd salvage, compared to 24% of adult patients [8]. Similarly, 43% of pediatric patients had undergone ≥1 HSCT prior to InO, vs. only 14% of adult patients. The adult experience with gemtuzumab ozogamicin suggests higher risk of SOS if the agent is followed by HSCT within 3 months [11]; however, this was not confirmed in a pediatric study [20]. The median time from last dose of InO to HSCT was 6 weeks in the adult InO study, and 3.7 weeks in this pediatric cohort; thus, it is not known if the risk of SOS with InO can be attenuated by delaying HSCT. We could not discern if higher pre-HSCT transaminase and bilirubin values were associated with a higher risk of SOS as these data were not collected, but there was no correlation of SOS to the occurrence of these toxicities during InO therapy. The use of two alkylators and/or busulfan in the conditioning regimen increased the risk of SOS in adult patients. Due to the small number in the pediatric cohort who underwent HSCT, we were unable to identify significant risk factors for SOS; however, there was a trend of developing post-HSCT SOS when conditioning included clofarabine and/or busulfan. Both patients with fatal SOS had received clofarabine for conditioning, an agent known to be associated with a high incidence of hepatotoxicity and SOS [18]. In addition, the more frequent use of myeloablative conditioning in pediatric patients compared to reduced intensity conditioning in adult patients may influence the incidence of post-HSCT SOS. The use of ursodiol or defibrotide for SOS prevention or treatment was not ascertained in a uniform manner. The use of these agents for prophylaxis may decrease the risk and severity of SOS during post InO HSCT, and therefore patients who undergo HSCT post InO are eligible for the ongoing study of defibrotide prophylaxis (NCT02851407). With improved access to CAR T-cell therapies more pediatric patients could potentially avoid a second HSCT and subsequent SOS.

This study has several limitations. Since only approximately 50% of patients who received InO by the compassionate use program as of the study cutoff date are included in this study, the introduction of unintended reporting bias could not be avoided. Additionally, it is a retrospective study wherein all data were reported by individual institutions that may have varied in the comprehensiveness of toxicity reporting; thus, positive findings may be more reliable than negative findings. However, we believe that major toxicities and grading were accurately captured. Unlike a prospective study with well-defined eligibility criteria, the heterogeneity of the patient cohort with respect to disease burden, performance status, comorbid conditions and number and type of prior therapies may impact interpretation of the results.

Despite these limitations, we show that InO is an effective and relatively safe agent for pediatric patients with relapsed/refractory ALL. There are currently two prospective pediatric phase II trials of InO for patients with relapsed/refractory, one in the United States (NCT02981628) and one in Europe (EudraCT 2016–000227–71); the latter is planned in combination with chemotherapy after the single agent phase. Data generated from these studies will provide more comprehensive data on the efficacy and toxicities of InO as it is potentially moved to frontline therapy. InO is a particularly attractive candidate for combination chemotherapy/immune-targeted therapy in de novo ALL, as the majority of these patients will not require HSCT, thus minimizing the risk of SOS which is the principal toxicity of concern with this agent.
