# Correction: Inotuzumab ozogamicin in pediatric patients with relapsed/refractory acute lymphoblastic leukemia

**DOI:** 10.1038/s41375-019-0426-8

**Published:** 2019-04

**Authors:** Deepa Bhojwani, Richard Sposto, Nirali N. Shah, Vilmarie Rodriguez, Constance Yuan, Maryalice Stetler-Stevenson, Maureen M. O’Brien, Jennifer L. McNeer, Amrana Quereshi, Aurelie Cabannes, Paul Schlegel, Claudia Rossig, Luciano Dalla-Pozza, Keith August, Sarah Alexander, Jean-Pierre Bourquin, Michel Zwaan, Elizabeth A. Raetz, Mignon L. Loh, Susan R. Rheingold

**Affiliations:** 1 Children’s Hospital Los Angeles and Keck School of Medicine, University of Southern California, Los Angeles, CA, USA; 2 National Cancer Institute, Bethesda, MD, USA; 3 Mayo Clinic, Rochester, MN, USA; 4 Cincinnati Children’s Hospital Medical Center, University of Cincinnati School of Medicine, Cincinnati, OH, USA; 5 University of Chicago Medicine Comer Children’s Hospital, Chicago, IL, USA; 6 Oxford University Hospitals, Oxford, UK; 7 University Hospital Saint-Louis, Paris, France; 8 University Children’s Hospital of Würzburg, Würzburg, Germany; 9 University Children’s Hospital of Münster, Münster, Germany; 10 Children’s Hospital at West-mead, Sydney, Australia; 11 Children’s Mercy Hospital, Kansas City, MO, USA; 12 The Hospital for Sick Children, Toronto, Canada; 13 University Children’s Hospital, Zurich, Switzerland; 14 Erasmus MC, University Medical Center, Rotterdam, the Netherlands; 15 New York University Langone Medical Center, New York, NY, USA; 16 Benioff Children’s Hospital and the Helen Diller Family Comprehensive Cancer Center, University of California San Francisco, San Francisco, CA, USA; 17 Childrens Hospital of Philadelphia, University of Pennsylvania, Philadelphia, PA, USA

In the original version of this Article the following authors were omitted:

- Claudia Rossig (University Children’s Hospital of Münster, Münster, Germany)
- Paul Schlegel (University Children’s Hospital of Würzburg, Würzburg, Germany)
- Jean-Pierre Bourquin (University Children’s Hospital, Switzerland)
- Aurelie Cabannes (University Hospital Saint-Louis, Paris, France)
- Luciano Dalla-Pozza (Children’s Hospital at West-mead, Sydney, Australia)
- Sarah Alexander (The Hospital for Sick Children, Toronto, Canada)
- Michel Zwaan (Erasmus MC, University Medical Center, Rotterdam, the Netherlands)
- Amrana Quereshi (Oxford University Hospitals, UK)
- Keith August (Children’s Mercy Hospital, Kansas City, MO, USA)

All authors agree to the inclusion of these added authors

